# Self- care practices and associated factors among adult diabetic patients in public hospitals of Dire Dawa administration, Eastern Ethiopia

**DOI:** 10.1186/s12889-020-09338-5

**Published:** 2020-08-12

**Authors:** Asmare Getie, Biftu Geda, Tadesse Alemayhu, Agenehu Bante, Zeleke Aschalew, Biresaw wassihun

**Affiliations:** 1 College of Medicine and Health Sciences, Arba Minch University, Arba Minch, Ethiopia; 2 School of Nursing and Midwifery, Department of public health, College of Health and Medical Sciences, Haramaya University, Harar, Ethiopia

**Keywords:** Diabetes mellitus, Self-care practice, Dire Dawa

## Abstract

**Background:**

Diabetes is a huge growing problem, and causes high and escalating costs to society. Self- care practice for adults with diabetes is not well addressed in sub-Saharan Africa including Ethiopia. To prevent serious morbidity and mortality, diabetes treatment requires a commitment to demanding self-care practice. This study aimed to assess self- care practices and its associated factors among adults with diabetes in Dire Dawa public hospitals of Eastern, Ethiopia.

**Methods:**

A cross-sectional study was conducted among 513 adults with diabetes. The study participants were selected through systematic random sampling. Data were collected from February 1st to March 1st, 2018. Patients were interviewed using a structured questionnaire. Data were entered into Epi-data version 3.3.1 and exported to SPSS version 22.0 for analysis. Bivariable and multivariable logistic regression with crude and adjusted odds ratios along with the 95% confidence interval was computed and interpreted accordingly. Good self-care was defined based on mean calculation; a result above the mean value had a good self-care practice, and a *P*-value of < 0.05 was considered to declare a result as statistically significant.

**Result:**

The result of the study showed that 55.9%, (95% CI: 51.4, 60.3) of participants had good self-care practices. Good self-care practice was associated with having family support, treatment satisfaction, diabetes education, having glucometer, higher educational status, duration of the disease, high economic status, and having good knowledge. Self-care practice was significantly associated with good diabetes knowledge (AOR = 2.14, 95% CI: 1.37, 3.35), family support system (AOR = 2.69, 95% CI:1.56, 4.62), treatment satisfaction (AOR = 2.07, 95% CI:1.18, 3.62), diabetes education (AOR = 2.21, 95% CI: 1.35, 3.63), high economic status (AOR = 1.89, 95% CI: 1.01, 3.48), having glucometer,(AOR = 2.69, 95% CI:1.57, 4.63),higher educational status (AOR = 2.68, 95% CI: 1.31, 5.49), and duration of disease greater than 10 years AOR = 2.70, 95% CI: 1.17, 6.26).

**Conclusion:**

In this study, a large number of adults had poor self-care practices which are very significant in controlling diabetes. Providing diabetes education, about self-care practices to the respondents and their families should be considerable.

## Background

Self-car is an individual’s adherence to a diabetes self-management regime, which is imperative to maintain glycemic control all-encompassing of diet, maintaining physical activity, daily monitoring of blood glucose levels, adhering to medication therapy and foot care [1]. Self-care is considered crucial for all people with diabetes for monitoring the disease process, prevention of complications, and glycemic control which improved quality of life [2].

Globally, an estimated 422 million adults were living with diabetes in 2014 [3]. Two-thirds of the global diabetes population lives in the developing world [4]. Based on the IDF Atlas 9th edition, the number of cases of diabetes globally was 463 million and Ethiopia was estimated at 1.7 million in 2019 [5]. Diabetes is one of the most challenging health problems in the twenty-first century. It costs at least 548 billion dollars in health expenditure in 2013 which is 11% of total health spending on adults [6]. When it is not prevented and properly managed, diabetes is one of the major causes of premature illness and death worldwide which resulted in 5.1 million deaths in 2013. Poor self-care practice increases the incidence and prevalence of complications resulting in increased morbidity, and mortality [7] To reduce morbidity and mortality different studies were conducted in different parts of the world. Although there was significant variation across countries, self-care practice on diabetes is less than optimal in all countries. More than one-third of US adults with diabetes have poor self-care practices [8]. Diabetic’s self-care practices in Ethiopia is still low, which is in the range of 39–63.3% [9]. Despite the great strides that have been made in the treatment of diabetes in recent years, many patients do not achieve optimal outcomes and still experience devastating complications due to inadequate self-care practice, and there is little information in the study area. Hence the finding of the study provides and fills the gap-related to the level of self-care practice. Therefore, this study aims to assess self-care practices and its associated factors among adults with diabetes in Dire Dawa city Administration Eastern Ethiopia.

## Methods

### Study area and period

The study was conducted in Dire Dawa Administrative city from the1st of February to the 1st of March 2018. Dire Dawa is located in the Eastern part of the country enclosed by Ethiopian Somalia and the State of Oromia. It is 515 km from Addis Ababa and 47 km from Harar town. It has a hot temperature with a mean of 25 degrees centigrade. Based on the 2007 Census, the total population of Dire Dawa Administrative city is 395,000 of which females account 51.6%.

It has 9 urban kebeles (small administrative units of Ethiopia) and 38 rural kebeles. The City has given health care services for the catchment population in different disciplines. The hospitals have provided services for adults with diabetes in the diabetic clinic. The service is given by physicians, and 2171 adults with diabetes were registered for follow-up in the past one year.

### Study design

Institutional based cross-sectional study design was conducted.

### Inclusion and exclusion criteria

#### Inclusion criteria

All adults with diabetes who had one year follow up and came into diabetic clinics were included in the study.

#### Exclusion criteria

Those adults with diabetes who are critically and mentally ill, pregnant women’s and newly diagnosed adults with diabetes were excluded.

#### Sample size determination and sampling procedures

The required sample size is determined by using a formula for single population proportion by taking different *p* values from different studies; and the sample size for some of the factors for diabetic self-care practices obtained from different pieces of literature was calculated by Epi Info 7 menu statically, by considering the following assumptions: confidence level 95%, power 80% and exposed to the unexposed ratio of 1. So, the sample size for this study was 466 and after adding a non-response rate of 10% the final sample size became 513.

#### Sampling procedures

In the study area there are two governmental hospitals (SGH=Sabian General Hospital and DCRH = Dill Chora referral hospital) and these two governmental hospitals were selected purposively. These two hospitals have separate clinics for diabetes follow-up. The number of study participants from the selected health facilities was determined from the previous total number of diabetic patients who have followed up which is 2171 in the two hospitals.

Samples were allocated to each of the selected hospitals based on proportional allocation to sample size. The lists of respondents or sampling frames were obtained from the updated registration books of each follow-up clinic of the hospitals. After establishing the sampling frames of respondents, a simple random sampling technique was used to identify the study unit to be included in the study. Adults with Diabetes were selected by computer method using updated registration books as a sampling frame until a sample of 513 was reached.

### Data collection tools

Six trained nurses conducted the interview using a structured pretested questionnaire for one month. The principal investigator and an assistant supervised the data collection. Face to face interviewer-administered validated questionnaire was used to measure self-care practice, which was contextualized to the study area. It was prepared originally in English and was translated to Amharic, and then translated back to English for checking the consistency of the questionnaire. The questionnaire consisted of socio-demographic variables (8 items), health-related variables (14 items), diabetic knowledge (15 items), treatment satisfaction (6 items), and self-care practice (15 items).

### Data collection technique

The data collection was conducted in an institution-based exit interview by using pre-tested Amharic, and English questionnaires. The training was provided for the data collectors and supervisors by the principal investigator. All adults with diabetes who are selected and fulfill the inclusion criteria were interviewed. Confidentiality and privacy were kept.

### Data analysis

The collected data were cleaned, coded, and entered into Epi Data 3.3.1 statistical software package. The statistical analysis was done using SPSS version 22. Frequency distribution for selected variables was done. The statistical significance and strength of the association between independent variables and an outcome variable were measured by the bivariate logistic regression model. A variable *P*-value less than 0.2 was candidates to multivariable logistic regression model and a *p*-value less than 0.05 was considered as significantly associated. Finally, the results of the study were presented using tables, figures, and texts based on the data obtained.

### Operational definitions

#### Self-care practices

It is the practice of activities that individual diabetics will initiate and perform on their behalf in controlling their disease, maintaining life, health, and wellbeing [10].

#### Good self-care practices

individuals who have scored mean and above the mean value of the total 15 self-care practice questions. These 15 items evaluate the status of patients’ self-care during the last seven days. Responses in each subscale were based on 7-days, ranged from 0 to 7, the higher number was indicative of days reflecting better self-care operation. The minimum score is 0 and the maximum score in this tool is 105, which is indicative of the highest quality of self-care.

#### Knowledge about diabetes

This is measured by fifteen items in yes/no format. The correct answer will be given “1” and “0” is given for incorrect and don’t know. Then a total score is computed out of fifteen marks (with the range of 0–15) those who score mean and above the mean have a good knowledge whereas those who score below the mean value have no good knowledge [11].

## Results

### Socio-demographic characteristics of the participants

A total of 513 diabetic patients were approached, among which all, but 7 consented and complete the interview. The seven interviewees consented, but could not finish the interview, giving a 98.6% response rate. From the total respondents, 279 (55.1%) were females and 227(44.9%) were males. More than half of the respondents (56.7%) were Orthodox, 170(33.6%) Muslims, and 47(9.3%) Protestants, regarding ethnicity 217 (42.9%) were Amhara and 190 (37.5%) were Oromo. More than half of the respondents (54.5%) were married, 71(14%) single, 53 (10.5%) widowed, and 106(20.9) were separated. The Mean age of the respondents was 51.48 (SD ± 14.75) years with a range of 18 to 86 years. From the total respondents one hundred seventy-two (34%) were self-employed (Table 1).

**Table 1:** Socio-demographic data of adult diabetic patients in Dire Dawa public hospitals 2018, (*n* = 506*)*

Characteristics	Alternative response	Frequency	
		No	%
Sex	Male	227	44.9
	Female	279	55.1
Ethnicity	Amhara	217	42.9
	Oromo	190	37.5
	Tigre	17	3.4
	Gurgie	53	10.5
	Somali	29	5.7
Religion	Orthodox	287	56.7
	Muslim	170	33.6
	Protestant	47	9.3
	Other	2	.4
Marital status	Married	276	54.5
	Single	71	14
	Widowed	53	10.5
	Separated	106	20.9
Level of education	Cannot read cannot write	130	25.7
	Read and write	70	13.8
	Primary school	101	20
	Secondary school	120	23.7
	College and above	85	16.8
Occupation	Self-employed	172	34
	Employed	105	20.8
	Unemployed	161	31.8
	Student	26	5.1
	Housewife	42	8.3
Wealth index	Low	170	33.6
	Medium	193	38.1
	High	143	28.3

### Health status of the study participants

The majority of the respondents, 378 (74.7%) were type 2 diabetic patients, 60.7% of the respondents had no history of co-morbidities, and most of the patients (61.3%) were on oral hypoglycemic agents and 24.3% were on insulin medication.

**Fig. 1:**
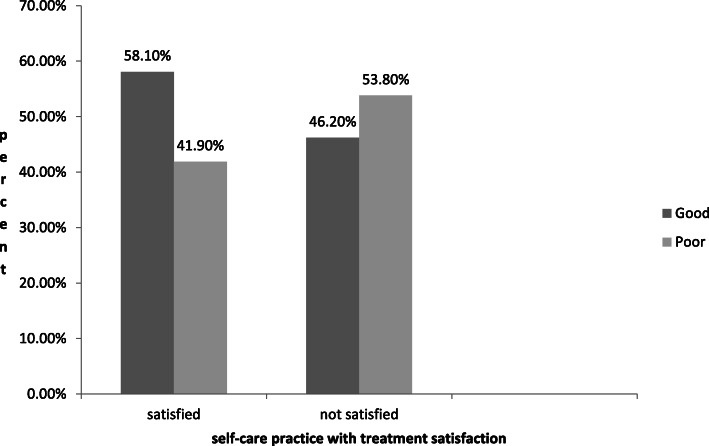
Self-care practice distribution with treatment satisfaction among adult diabetic patients in Dire Dawa public hospitals Eastern Ethiopia, 2018

Only 34 (6.7%) of the participants were members of the Ethiopian Diabetes Association. The majority of the participants (77.5%) had family support. About 231 (45.7%) of the participants usually received diabetes education and more than half of the participants (53.6%) were knowledgeable. The diabetic treatment satisfaction rate was 81.6% (Fig. 1**).**

From the total respondents, one hundred fifty-one (29.8%) had a family history of diabetes and nearly one-fifth of the respondents (18.8%) had glucometer at home. More than one-third of the participants 199 (39.3%) had long term diabetic complications confirmed medically and almost all 187(94%) have hypertension (Table 2).

**Table 2:** Health status data of type 2 diabetes patients in Dire Dawa public hospitals, 2018 (*n* = 506)

Characteristics	Alternative response	Frequency	
		No	%
Knowledge of diabetes	Good	271	53.6
	Poor	235	46.4
Duration of diabetes	1–5 years	215	42.5
	6–10 years	240	47.4
	Greater than 10 years	51	10.1
Comorbidities	Yes	199	39.3
	No	307	60.7
Current treatment	Oral	310	61.3
	Insulin	123	24.3
	Both	73	14.4
Having glucometer	Yes	93	18.38
	No	413	81.62
Getting DM education	Yes	231	45.7
	No	275	44.3
Members of Diabetes association	Yes	34	6.7
	No	472	93.3
Treatment satisfaction	Yes	413	81.6
	No	93	18.4
Having family support	Yes	392	77.5
	No	114	22.5
Having support from society	Yes	19	3.8
	No	487	96.2
Alcohol drinking	Yes	35	6.9
	No	471	93.1

### The magnitude of self care practices

Overall self-care practices were calculated using 15 self-care assessment tools with a total score of 105. Using this, the overall mean score for self-care was 43.5(SD ± 10.9) and range from19–69 points. Two hundred eighty-three subjects (55.9%) scored above the mean value from the total self-care questions. As a result, 55.9%, (95% CI: 51.4, 60.3) have good self-care practices.

Regarding adherence to regular exercise, 294 (58.1%) of the respondents adhered to the recommended daily regular exercise; nearly more than half (53.8%) of the respondents practiced a physical activity of at least 30 min on all days of the week and twenty-five (5%) had A separate exercise session more than 3 days per week apart from their day to day physical activities.

Regarding medication, the majority (91.7%) of the study participants adhered to the prescribed medication on all days of the week. The majority, (78.5%), of the respondents, didn’t practice the recommended self -monitoring of blood Glucose which means, monitoring their blood sugar level less than the mean value.

More than half (54.9%) of the study participants did not adhere to the recommended diet management practices which means below the mean value. Around two-thirds (62.1%) of the respondents had practiced the recommended diabetic foot care, which scored mean and above the mean. From the total respondents, 386 (76.3%) had washed their feet all days of the week, 273 (54%) of diabetic patients had checked their feet on all days of the week. More than half, (55.7%), of the respondents, performed daily foot inspection, and nearly greater than half (51%) of respondents reported that they dried between their toes after washing their feet (Table 3).

**Table 3:** Self-care practices among adults with diabetes and factors associated with self-care in Dire Dawa public hospitals, 2018

Variables	Self-care practice		COR; 95% CI	AOR; (95% CI
	Good N (%)	Poor N (%)		
**Duration of disease in Year**				
1–5	16(39)	25(61)	1.00	1.00
6–10 years	90(51.75)	84(48.3)	0.146(0.84–3.35)	1.56(0.99–2.46)
> 10 years	177(60.8)	114(39.2)	2.426(1.24–4.74)	3.339(1.51–7.38)
DM Education				
Received	153(66.2)	78(33.8)	2.188(1.52–3.13)	2.20(1.34–3.62)*
Not received	130(47.3)	145(52.7)	1.00	1.00
**Sex**				
Male	143(63)	84(37)	1.00	1.00
Female	140(50.2)	139(49.8)	1.69(1.182–2.416)	0.906(0.55–1.48)
**Disease knowledge**				
Good	188(69.4)	83(30.6)	3.338(2.31–4.81)	2.144(1.37–3.34)*
poor	95(40.4)	140(59.6)	1.00	1.00
**Glucometer at home**				
Yes	78(83.9)	15(16.1)	5.27(2.93–9.47)	2.69(1.56–4.62)*
No	205(49.6)	208(50.4)	1.00	1.00
**Mode of treatment**				
Tablet	167(53.9)	143(46.1)	1.00	1.00
Insulin	116(59.2)	80(40.8)	0.80(0.56–1.15)	0.97(0.61–1.54)
**Educational level**				
Not read and write	62(47.7)	68(52.3)	1.00	1.00
Read and write	26(37.1)	44(62.9)	0.64(0.35–1.17)	0.77(0.36–1.63)
Elementary school	58(57.4)	43(42.6)	1.48(0.87–2.49)	1.55(0.75–3.17)
Secondary school	74(61.7)	46(38.3)	1.764(1.066–2.92)	1.95(0.95–3.95)
College and above	63(74.1)	22(25.9)	3.14(1.73–5.69)	2.70(1.17–6.25)*
**Marital status**				
Married	162(58.7)	114(41.3)	1.53(0.97–2.49)	0.77(0.42–1.39)
Single	44(62)	27(38)	1.75(0.78–3.21)	0.859(0.36–2.00)
Widowed	26(49.1)	27(50.9)	1.038(0.53–2.00)	0.747(0.33–1.69)
Separated	51(48.1)	55(51.9)	1.00	1.00
**Family support**				
Having	246(62.8)	146(37.2)	2.28(1.52–3.43)	2.69(1.56–4.62)*
Not having	37(32.5)	77(67.5)	1.00	1.00
**Treatment satisfaction**				
Satisfied	240(58.1)	173(41.9)	1.61(1.02–2.53)	2.06(1.18–3.61)*
Not satisfied	43(46.2)	50(53.8)	1.00	1.00
**Wealth index**				
Low	76(44.7)	94(55.3)	1.00	1.00
Medium	123(63.7)	70(36.3)	2.1(1.42–3.31)	1.47(0.85–2.53)
High	84(58.7)	59(41.3)	1.76(1.12–2.76)	1.88(1.02–3.47)

*Statistically significant at *P* < 0.05

## Discussion

In this study, the current self-care practices of peoples with diabetes in Dire Dawa public hospitals and factors that contribute to the self-care practice of diabetics were investigated. The finding of this study was addressed factors that were not well studied by other studies like foot care practice and it provides pertinent information to the population in the study area as well for the scientific society since there were no previous local studies. Overall, Self-care practices among people with diabetes were found to be good at 55.9%, (95% CI: 51.4, 60.3) of the study participants.

The predictors of self-care practices were: getting an education from health professions, having glucometer at home, having knowledge about diabetes, treatment satisfaction, duration of disease, having family support, educational status, and family wealth index.

The overall recommended self-care practice was (55.9%). This finding was in line with the results of the studies conducted in Addis Ababa (60.2%) [12]. However, the finding of this study was greater than the study conducted in Harar, Eastern Ethiopia (39.2%) [13]. This discrepancy may be due to some improvements in the health care systems (related to the period gap) and variation of cutoff point to classify good and poor self-care, mean, and 50 % of total self-care practices, respectively. Sample size variation may also attribute to this difference.

On the contrary, the finding of this study was lower than the study conducted in Eastern Nepal of which (70%), of the study participants, had good self-care practices [14]. This variation could be due to socio-cultural differences. The majority of the study participants in Nepal had a high income, so that they could afford their own glucometer and easily get a healthy diet. This finding is also lower than the study which was conducted in Dilla, South Ethiopia which had 76.8% good self-care practices. Methodological and sample size variation may also account for this discrepancy [11].

This study revealed that those who had family support were nearly three times more likely to have good self-care practices than those who did not. This is consistent with a study conducted in Addis Ababa [12] and Kenya [15]. Individuals who have family support could have better information related to disease, have a chance to get an education, and may have got a good income.

Those who had received education from health care professionals were almost two times more likely to have good self-care practice than those who didn’t. This finding was comparable with the study conducted in Addis Ababa [12] and Harari [13].

Respondents who had a higher level of education were nearly three times more likely to be engaged in self-care practices when compared with respondents who were unable to read and write. This finding was congruent with the studies conducted in Black Lion, hospital, and study conducted in Gondar [7, 12]. This implies that education is the base for a diabetic patient to understand the disease process and to provide own self-care practice, because they may be able to read and become more informed of the benefits of adherence.

Knowledge of diabetes and its disease process was found to be positively associated with self-care practice. Individuals who were knowledgeable were nearly two times more likely to have good self-care practice than those who had less diabetes knowledge. This finding was in line with the finding from Addis Ababa, Malaysia, and the Harari Region of Eastern Ethiopia [8, 12, 13]. The possible justification is that the right knowledge about diabetes and its self-care practice creates a clear understanding and avoids confusion about the practice and the disease condition.

Respondents who have glucometer at home were nearly three times more likely to have good self-care practice when compared to those who didn’t. This finding was in line with the finding of the study which was conducted in Black Lion Specialized Referral Hospital, Addis Ababa [4].

In this study participants who had high income were two times more likely to have good self-care practices than those who had low income. This finding was in line with the study conducted in Dilla University Referral Hospital and Arba Minch General Hospital [10, 11].

Respondents who were satisfied regarding treatment were nearly two times more likely to have good self-care practices than their counterparts. This finding was congruent with a study conducted in Nigeria [16]. Since patient satisfaction is directly associated with the degree of satisfaction with expected care and is linked with cognitive evaluation and emotional reactions to the components of care services.

## Conclusions

In this study, a large number of adults had poor self-care practices which are very significant in controlling diabetes. Providing diabetes education, about self-care practices to the respondents and their families should be considerable. Subjects who had no glucometer, lack of family support, low educational status, low diabetes education, low economic status, and poor diabetes knowledge were negatively associated with self-care practice.

The hospital administration should stress on educating clients more during follow up periods and diabetic self-care should be incorporated in the health education program of the hospitals.

Healthcare professionals should use precise and clear ways to provide information for respondents and their families to emphasis on dietary management.

Interventional studies are recommended to determine the outcome of the information provided on diabetes self-care practices of adults with diabetes.

## Data Availability

The datasets used and/or analyzed during the current study are available from the corresponding author on reasonable request.

## Abbreviations

DM: Diabetes mellitus: confidence interval
AOR: Adjusted odds ratio
IDF: International diabetes federation
SMBG: Self-monitoring of Blood Glucose
US: United States
